# Surface modification of commercial anion exchange membrane for the inactivation of *Escherichia coli*

**DOI:** 10.1007/s11356-026-37525-1

**Published:** 2026-02-24

**Authors:** Fulufhelo H. Mudau, Ralph F. Muvhiiwa, Machawe M. Motsa, Lueta-Ann De Kock, Francis Hassard

**Affiliations:** 1 College of Science, Engineering and Technology, University of South Africa, Johannesburg, 1709 South Africa; 2 Cranfield Water Science Institute, Cranfield University, Bedfordshire, MK43 0AL UK

**Keywords:** Water treatment, Antimicrobial efficiency, Anionic membranes, Intermatrix synthesis, Silver nanoparticles, Copper nanoparticles

## Abstract

Modifying membranes with antimicrobial nanoparticles enhances antifouling properties and enables rapid disinfection during water treatment. Here, silver (Ag) and copper (Cu) particles were formed on a commercial anionic exchange membranes using a two-step ion-mediated surface-reduction process consisting of a 24-h sodium borohydride treatment followed by a 24-h reaction with Ag and Cu precursor solutions (0.01–0.1 M). Scanning electron microscopy with energy-dispersive spectrometry confirmed uniform Ag and Cu particle distribution on the membrane surface. Increasing precursor concentration enlarged the Ag particle diameters from 167.7 ± 2.2 nm to 652.2 ± 23.4 nm and Cu from 117.8 ± 3.4 nm to 606.5 ± 16.6 nm, with metal content of 0.05 ± 0.001–0.17 ± 0.01 mg·cm^2^ (Ag) and 1.05 ± 0.01–2.13 ± 0.03 mg·cm^2^ (Cu). Metal leaching after 14 days was low (Ag: 3.11 ± 0.24–6.62 ± 0.12 ppb; Cu: 2.75 ± 0.1–5.32 ± 0.1 ppb), within World Health Organization drinking water limits. The modified membranes achieved up to 8-log inactivation of *Escherichia coli* within 1–4 h. The most effective Ag and Cu membranes (lowest metal loading) exhibited specific inactivation rates of 13.68 ± 0.93 (mg·cm^2^)⁻^1^ for Ag and 1.69 ± 0.14 (mg·cm^2^)⁻^1^ for Cu in 2 h. Ag exhibited the highest antimicrobial efficiency per unit metal, while high-loading Cu disinfects fastest, together showing metal-modified anionic membranes provide stable, low-leaching antimicrobial performance suitable emergency treatment.

## Introduction

Access to safe drinking water remains a global priority, particularly in decentralized and resource-limited regions where conventional disinfection technologies are difficult to maintain or deploy (Sombei et al. 2025). Waterborne pathogens, biofilm formation, and persistent microbiological contamination continue to undermine the safety of small-scale and emergency water treatment systems (Li et al. 2024). As a result, there is a growing interest in developing passive, material-based disinfection technologies that can provide reliable microbial inactivation without continuous chemical additions or complex operational control.

Industrial polymeric membranes play a critical role in modern water treatment due to their high separation performance, mechanical strength, and scalability (Sahu et al. 2024). However, most commercial polymers, including polyvinyl chloride, polysulfone, and ion exchange membranes, lack intrinsic antimicrobial activity and are prone to biofouling (Sahu et al. 2024). Consequently, extensive research has focused on modifying the surface chemistry and functional groups of industrial polymers to enhance reactivity and contaminant-removal performance (Hao et al. 2023). For example, recent work on polyvinyl chloride–based cation exchange resins demonstrated how sulfonation and functional group incorporation can significantly improve ion exchange efficiency and water softening capacity (Mukhamediev et al. 2021). Bio-based polymer materials such as cellulose and starch composites have also been engineered with active sites or metal additives to enhance adsorption, decontamination, and microbial removal in wastewater treatment (Bekchanov et al. 2024).

Advances in hybrid and nanocomposite materials have further demonstrated the potential of integrating metals and metal oxides into polymer frameworks. Silver (Ag)-based hybrid structures such as Ag/AgCl@MIL-53(Fe) have shown strong photocatalytic and antimicrobial properties due to synergistic interactions between plasmonic silver and semiconductive frameworks, resulting in efficient generation of reactive oxygen species and enhanced pollutant degradation (Liu et al. 2024). Similarly, polymer–metal oxide nanocomposites incorporating materials such as TiO_2_, ZnO, or Fe_3_O_4_ exhibit high antibacterial performance and photocatalytic activity, driven by optimizing nanoparticle dispersion, polymer computability, and surface interactions (Bekchanov et al. 2025). Collectively, these advances underscore the value of developing multifunctional polymer–nanoparticle materials capable of simultaneous disinfection, fouling resistance, and pollutant removal.

Recent progress confirms that the modification of ion exchange membranes with metal nanoparticles is a promising strategy for developing hybrid materials with enabling built-in antimicrobial functionality during filtration and water treatment applications. Silver (Ag) and copper (Cu) nanoparticles, in particular, offer a broad spectrum of antimicrobial activity through membrane disruption, oxidative stress, metabolic interference, and control of metal–ion release (Godoy-Gallardo et al. 2021). Their incorporation into polymer matrices has been shown to suppress biofilm, enhance membrane durability, and support decentralized disinfection systems (Nath et al. 2024). However, challenges remain in controlling nanoparticle formation, distribution, and stability on commercial membranes (Abounahia et al. 2022). Excessive agglomeration, inconsistent loading, and uncontrolled leaching can compromise both antimicrobial performance and drinking water safety (Mahlangu et al. 2024).

Intermatrix synthesis (IMS) provides an effective approach for generating nanoparticles directly within or on the surface of ion exchange polymer using charged functional groups to mediate particle formation and anchoring (Bastos-Arrieta et al. 2016a, b). By tuning precursor concentration and reduction conditions, IMS allows for precise control of nanoparticle size, localization, and stability, properties essential for safe and efficient antimicrobial membranes (Bastos-Arrieta et al. 2016a, b). Despite its potential, limited research has focused on applying IMS in commercially available anion exchange membranes and evaluating the interplay between nanoparticle characteristics, membrane chemistry, and antimicrobial efficacy.

In this work, commercial anion exchange membranes (AMI 7001) were modified using a two-step ion-mediated surface-reduction process to generate Ag and Cu nanoparticles directly on the membrane surface. The effect of metal precursor concentration on nanoparticle morphology, surface distribution, and loading, as well as the long-term stability of the membrane was evaluated. Finally, the antimicrobial performance against Escherichia coli (*E. coli*) was assessed, providing insights into the mechanisms governing nanoparticle-mediated disinfection and their potential application of metal-modified anion exchange in decentralized or emergency water treatment systems.

## Materials and methods

### Chemicals

A commercial heterogeneous anion exchange membrane (AMI 7001, Membrane International Inc. USA) was used to fabricate Ag and Cu-modified membranes. Silver nitrate (AgNO_3_, ≥ 99%), copper sulfate (CuSO_4_, ≥ 99%), and sodium borohydride (NaBH_4_, ≥ 98%) purchased from Sigma Aldrich (UK) were used for the synthesis of metal-modified membranes. Tryptone soya broth (TSB) (Oxoid, UK) and ringer solution ¼ strength tablets (Sigma Aldrich, UK) were used for culture maintenance and inoculum preparation just prior to bactericidal testing experiments.

### AgNP and CuNP anion exchange membrane synthesis

The IMS of AgNPs and CuNPs in the membrane was carried out by a two-step procedure (Bastos-Arrieta et al. 2015). In detail, a piece of membrane (5 cm × 5 cm) was immersed for 24 h in 20 mL of a solution of 0.1 M NaBH_4_ (pH 10.4), which exchanged the chloride counter ion of the exchange groups with the reducing agents (Eq. 1). Next, the membrane sections were treated with 20-mL solutions of AgNO_3_ (pH 5.9–6.2) (Eq. 2) or CuSO_4_ (pH 4.3–4.8) (Eq. 3). The reactions (detailed in Eq. 2 and Eq. 3) were allowed to proceed for 24 h, which resulted in the formation of metal nanoparticles on the surface of the membrane. A range of different metal precursor concentrations was loaded on the membrane (Table 1). The nanocomposite membranes were rinsed with deionized water, air dried, and then heated (60°C) for > 60 min prior to subsequent experiments. The membranes were stored in the dark at 4 °C prior to use.

**Table 1:** Metal precursor concentrations loaded on the membrane

Sample	Ag (mol·L^−1^)	Cu (mol·L^−1^)
M-Ag1	0.01	
M-Ag2	0.025	
M-Ag3	0.05	
M-Ag4	0.1	
M-Cu1		0.01
M-Cu2		0.025
M-Cu3		0.05
M-Cu4		0.1

Reduce-loading stage

1 $$R-{(R}_{3}{N)}^{+}{Cl}^{-}+ {NaBH}_{4}\leftrightarrow R-{\left({R}_{3}N\right)}^{+}{BH}_{4}^{-}+ NaCl$$

Ag-loading-reduction stage

2 $$(R-({R}_{3}{N)}^{+}{BH}_{4}^{-})+{AgNO}_{3}+3{H}_{2}O\to (R-({R}_{3}{N)}^{+}{{NO}_{3}}^{-}){Ag}^{0}+{\frac{7}{2}H}_{2 }+B({OH)}_{3}$$

Cu-loading-reduction stage

3 $$2(R-({R}_{3}N{)}^{+}B{H}_{4}^{-})+CuS{O}_{4}+6{H}_{2}O\to (R-({R}_{3}N{){)}_{2}}^{+}S{O}_{4}^{2-})C{u}^{0}+ 7{H}_{2}+2B{\left(OH\right)}_{3}$$

### Nanocomposite characterization

A scanning electron microscope (SEM) (Joel IT 300 SEM, Tokyo, Japan) with an energy-dispersive spectroscopy probe and detector (EDS, Oxford Instruments, UK) was used to analyze the morphology and elemental composition of the membranes. All membrane samples were coated with gold (Quorum Q150R ES coater, Quorum Technologies, Laughton, UK) prior to SEM imaging. EDS was used to determine the elemental composition of the membranes. Identification of elements was performed using software analysis (AZtech software, Oxford Instruments, UK) and comparison with a reference library of spectral data.

The average hydrodynamic diameter of the powder Ag and Cu NPs and those embedded within the polymer matrix was measured through dynamic light scattering (DLS) using a Malvern Zetasizer nanoseries (Malvern Instruments Ltd., UK). Samples of the nanocomposite membranes were dissolved in N-methyl-2-pyrrolidone (NMP) and sonicated for 15 min. When the NPs had dispersed in the organic solvent, they were analyzed by the Malvern Zetasizer nanoseries. Experiments were carried out on three replicate membrane samples to determine the average size of the NPs.

The metal content of the nanocomposite membranes was assessed by immersing 1 cm × 1 cm sections of each membrane in 1-mL concentrated HNO_3_ (65% *w*/*w*, Sigma Aldrich, South Africa) for 24 h to completely dissolve all the metal nanoparticles (Domenech et al., 2014). The leachate was diluted 1:100 with distilled water. The diluted leachates (2 method replicates) were analyzed for Ag and Cu content by inductively coupled plasma atomic emission spectrometry (ICP-OES) (Agilent Technologies 700 Series ICP-OES). External calibration standards (0.2–5 mg·L^−1^) were prepared using MERCK Certipur 111355 ICP multielement standard solution IV containing elements Ag, Al, B, Ba, Bi, Ca, Cd, Co, Cr, Cu, Fe, Ga, In, K, Li, Mg, Mn, Na, Ni, Pb, Sr, Ti, and Zn at a concentration of 1000 mg·L^−1^. Double-deionized water was used as a negative control. Calibration was linear over the concentration range studied in all cases. The membrane metal content (MMNP) was calculated using (Eq. 4)

$$MMNP=\frac{C\times V\times DF}{area} (4)$$

where *C* = concentration of diluted sample (mg·L^−1^), *V* = volume of the acid/leachate (L), *DF* = dilution factor, and *area* = area of the nanocomposite membrane (cm^2^).

### Metal nanocomposite stability

The 2018 US EPA and WHO limits for Ag and Cu in drinking water was 0.1 mg·L^−1^ and 1.3 mg·L^−1^ respectively (EPA, 2018; WHO, 2017). To validate that the modified nanocomposite membrane may treat water and not release unacceptable amounts of Cu and Ag, the stability and leaching from the nanocomposite membranes were evaluated. Two sections of 1 cm × 1 cm of each nanocomposite membrane were immersed in 10 mL of deionized water and incubated for 7 and 14 days at 25 °C with mixing at 150 rpm. The amount of metal leached was determined by ICP-OES at days 7 and 14 (Agilent Technologies 700 Series ICP-OES), and the percentage of metal release was assessed (Eq. 5).

4 $$R\%=\frac{{C}_{\text{metal solution}}\times V}{{\text{metal content}}_{\text{ membrane}}\times {A}_{\mathrm{membrane}}} \times 100$$

where *R*% = release percentage (%), *C*_metal solution_ = metal content in the solution (mg·L^−1^), *V* = volume of leachate (L), metal content_membrane_ = original metal content in the membrane (mg·cm^−2^), and *A*_membrane_ = area of membrane used for leaching (cm^2^).

### Bactericidal testing

*Escherichia coli *(*E. coli* ATCC 15597 LGC, UK) was cultivated in sterilized tryptone soya broth (TSB) (Oxoid, UK) and incubated overnight at 37°C. Bacteria were harvested during the mid-exponential period, where the optical density at 600 nm (OD600) of the culture was approximately 1.0, measured at a wavelength of 600 nm by visible spectrophotometry (6300, Jenway, UK). The bacteria were separated from the nutrient broth by centrifuging for 5 min at 5000 xG, thrice rinsed, and then resuspended in filtered (0.22 µL) ringer solution ¼ strength (pH 7) (Sigma Aldrich). This culture was diluted to an initial absorption value of approximately 0.1, which corresponded to ~10^8^ colony-forming units (CFU)/mL. To determine the bactericidal properties of the modified membranes compared to the unmodified membrane, a sample of 1 cm^2^ of each membrane was immersed in 20 mL of bacterial suspension and maintained at 37 °C with gentle agitation (100 rpm). A 100-µL suspension was collected after 0.5, 1, 2, 3, 4, and 24 h and cultured in tryptic soy agar (TSA, pH 7.3) medium plates in triplicates. The nutrient plates were incubated at 37 °C for 24 h, and the colonies were counted using a colony counter. The inactivation rate of *E. coli* using Ag and Cu modified membranes was calculated using Eq. (5)

5 $$Log \frac{{C}_{t}}{{C}_{0}}=-kd\times \text{metal content}$$

where *C*_*t*_ = bacteria concentration at time *t* (CFU/mL), *C*_0_ = initial concentration of bacteria at time 0 (CFU/mL), *kd* = inactivation rate ((mg·cm^−2^)^−1^), and metal content of nanocomposite (mg·cm^−2^).

### Biofouling inhibition

To further understand the role of contact between the MNPs and target bacteria for inhibition of biofouling, the agar diffusion method was applied (Zhang et al., 2014). In detail, *E. coli* ATCC 15597 was inoculated into sterilized liquid TSB (pH 7.3) and incubated overnight at 37°C. The resulting cell suspension was diluted to approximately 10^8^ CFU/mL. Aliquots (100 µL) of the diluted working suspension inoculated with *E. coli* were applied to agar (TSA, Sigma Aldrich, SA) plates evenly. Membrane samples (diameter 2.1 cm) were then placed onto the nutrient agar plates with the selective layer in contact with the agar surface. After incubation at 37 °C for 24 h, the bacteria inhibition zone of each plate was observed. To assess the interaction between the modified membranes and bacteria, membrane samples from the agar plates were coated by sputtering gold and subsequently examined by SEM.

## Results and discussion

### Membrane characterization

**Fig. 1:**
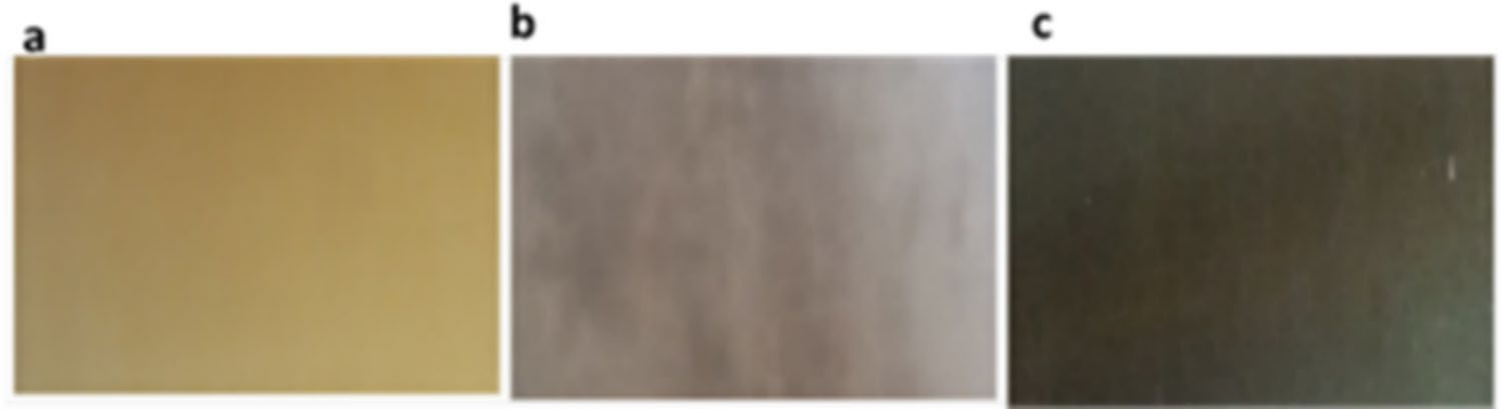
The photographic images of the membranes showing the color change after modification: **a** unmodified M-0, **b** Ag-modified membrane, and **c** Cu-modified membrane

A significant visual change was observed in the color of the membrane surface following the loading and reduction of Ag and Cu ions to their elemental forms. The Ag-modified membrane surface exhibited a gray color, while the Cu-modified membranes turned black (Fig. 1). This observation aligns with previous findings reported by Domènech et al. (2014), which noted that membranes darkened after the IMS procedure involving Ag and Pd loading (Domènech et al., 2014).

#### Morphological studies

**Fig. 2:**
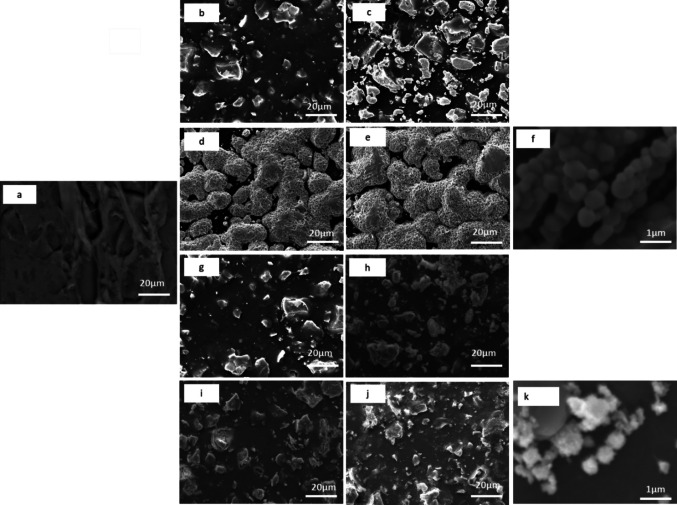
Scanning electron micrographs illustrating the surface of pristine and modified membranes containing silver (Ag) and copper (Cu): **a** M-0, **b** M-Ag1, **c** M-Ag2, **d** M-Ag3, **e** M-Ag4, **f** M-Ag4 at 2200× magnification, **g** M-Cu1, **h** M-Cu2, **i** M-Cu3, **j** M-Cu4, and **k** M-Cu4 at 2200× magnification

SEM analyses were performed to determine the distribution of Ag and Cu NPs after formation by IMS and the impact of this process on membrane morphology. The surface images of the pristine membrane and the Ag and Cu-modified membrane are shown in Fig. 2. The untreated membrane surface showed a uniform and dense morphology with no pores (Fig. 2a). The SEM imaging of Ag-modified membranes indicated that AgNPs were uniformly distributed on the surface of the membranes (Fig. 2b–e). As expected, the density of the AgNPs deposits increased as the concentration of the Ag precursor solution increased from 0.01 M to 0.1 M. For M-Ag1 and M-Ag2 membranes, the nanoparticles had flat plate-like structures on the surface (Fig. 2b, c). The M-Ag3 and M-Ag4 SEM images showed sponge-like structures on the surface (Fig. 2d, e). Aggregation of Ag NPs was observed for all Ag-modified membranes. The SEM micrograph of M-Ag4 at higher magnification (×2200) clearly shows that Ag particles (M-Ag4) are aggregated spheres (Fig. 2f).

Similarly, for the Cu-modified membranes, the CuNPs can be observed as a white deposit on the membrane surface (Fig. 2g–k). The SEM images showed that CuNPs are homogeneously distributed and embedded into the membrane matrix with flat plate-like morphology for all Cu-modified membranes. Increasing the concentration of the Cu precursor solution (0.01 M–0.1 M) influenced the number of NPs as more CuNPs were visible and distributed throughout the membrane matrix as precursor concentration increased. At higher magnification (×2200), the Cu particles of M-Cu4 appear spherical (Fig. 2k).

The morphological results are consistent with the IMS reactions outlined in Eqs. 1 to 3 in Section 2. These results demonstrate that both the membrane matrix and the metal ions possess a positive charge, leading to electrostatic repulsions that hinder the diffusion of metal ions into the membrane matrix. These observations indicate that the Ag and Cu particles are predominantly concentrated on the membrane surface. The BH4 − ions, which are incorporated through ion exchange and bonded to the quaternary ammonium functional groups, facilitate the reduction of the metal ions. Thus, the elemental distribution of Ag and Cu can be attributed to the widespread presence of quaternary ammonium groups on the membrane surface. This finding aligns with the work of Jiang et al. (2022), which links the distribution of Ag and Cu to the sulfonate groups present in the membranes (Jiang et al. 2022). An increase in the number of Ag and Cu particles, as well as their aggregation per unit area, is anticipated with rising concentrations. The increased aggregation occurs because higher concentrations lead to an excess of Ag and Cu ions, creating more opportunities for nucleation and growth sites, resulting in greater particle density and subsequent aggregation (Wagh et al. 2023). It is noteworthy that prior studies (Alonso et al. 2013; Bastos-Arrieta et al. 2013; Domènech et al. 2013) reported the formation of non-aggregated metal nanoparticles. However, our study observed greater aggregation corresponding to the increased precursor solution, which can also be attributed to the higher density of quaternary ammonium groups per unit area on the membrane influencing the growth sites (Abu Saleem et al. 2019).

**Fig. 3:**
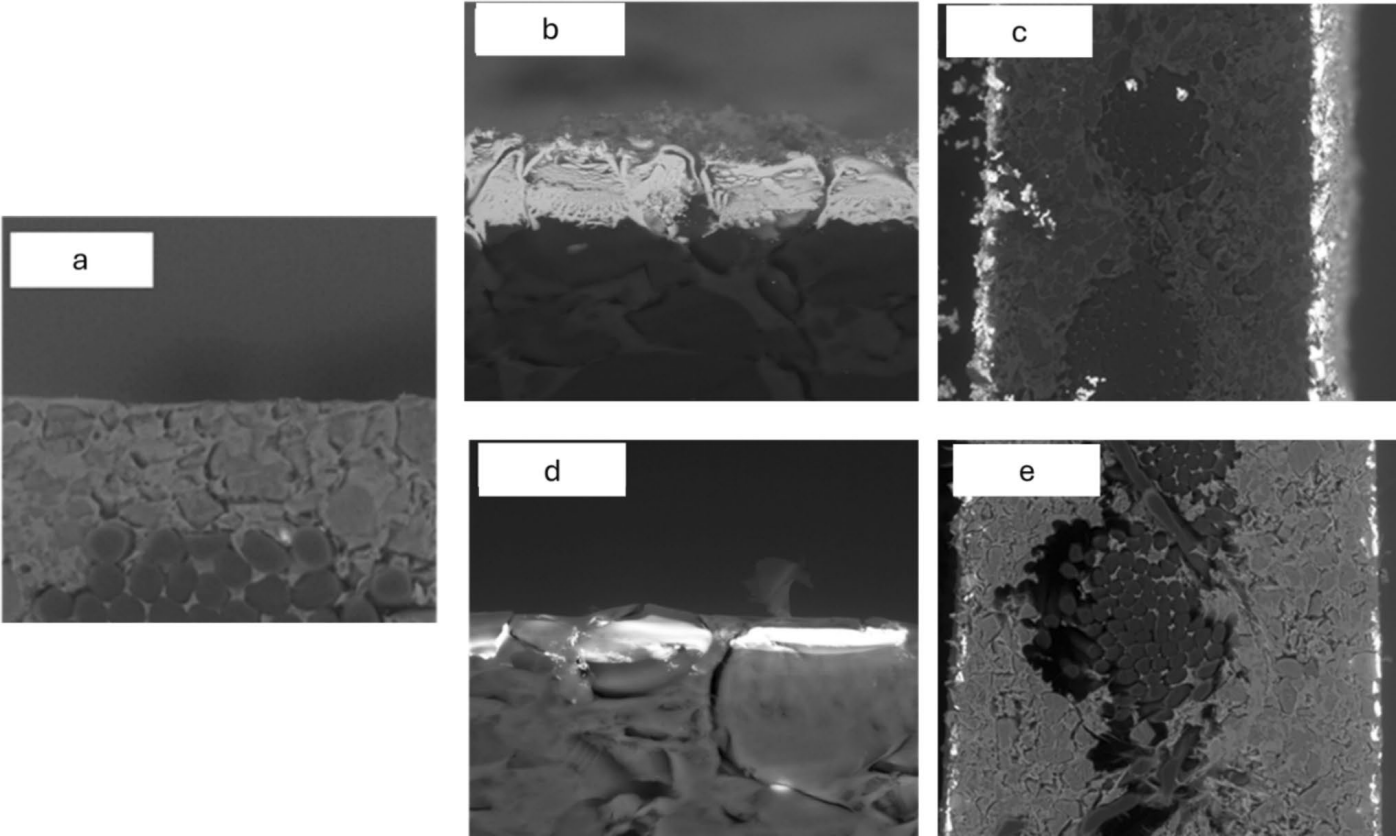
SEM micrographs showing the cross section of pristine M-0 (**a**), Ag-modified M-Ag4 (**b**, **c**), and Cu-modified M-Cu4 (**d**, **e**) membranes

The SEM cross-sectional analysis of the modified membrane (M-Ag4) confirmed that the AgNPs are concentrated mainly on the membrane surface with little intrusion into the polymer matrix (Fig. 3b). The Cu-modified membrane (M-Cu4) cross-sectional image indicates that CuNPs are embedded inside the membrane surface (Fig. 3c). The untreated membrane shows no layer deposition (Fig. 3a). The cross-sectional analysis of both Ag- and Cu-modified membranes reveals the presence of particle deposition on the surface (selective layer) and the bottom layer; however, no particles are detected within the membrane matrix (see Fig. 3c, e). These observations can be attributed to the coupling of the IMS procedure with the Donnan exclusion effect (DEE), which prevents Ag and Cu ions from penetrating deeply into the membrane matrix due to electrostatic repulsion (Mudau et al. 2023). Variations in the mobility and ionic radii of the metal ions may account for differences in membrane morphology (Domènech et al., 2014b). Consequently, the permeability of ions through a membrane matrix is closely linked to their hydrated radii; ions with smaller ionic radii generally exhibit higher hydration numbers and larger hydrated radii, facilitating easier permeation through the membrane. Specifically, Cu^2^⁺ ions can permeate the membrane surface more readily due to their smaller ionic radius (73 pm) in contrast to Ag⁺ ions (115 pm) (Saito et al. 2016). As a result, Ag particles tend to be more concentrated on the surface, while Cu is more embedded within the membrane surface. Nonetheless, the positioning of Ag and Cu particles on or near the membrane surface significantly enhances their biocidal efficiency and helps prevent biofilm formation (Fontecha-Umaña et al. 2020).

**Fig. 4:**
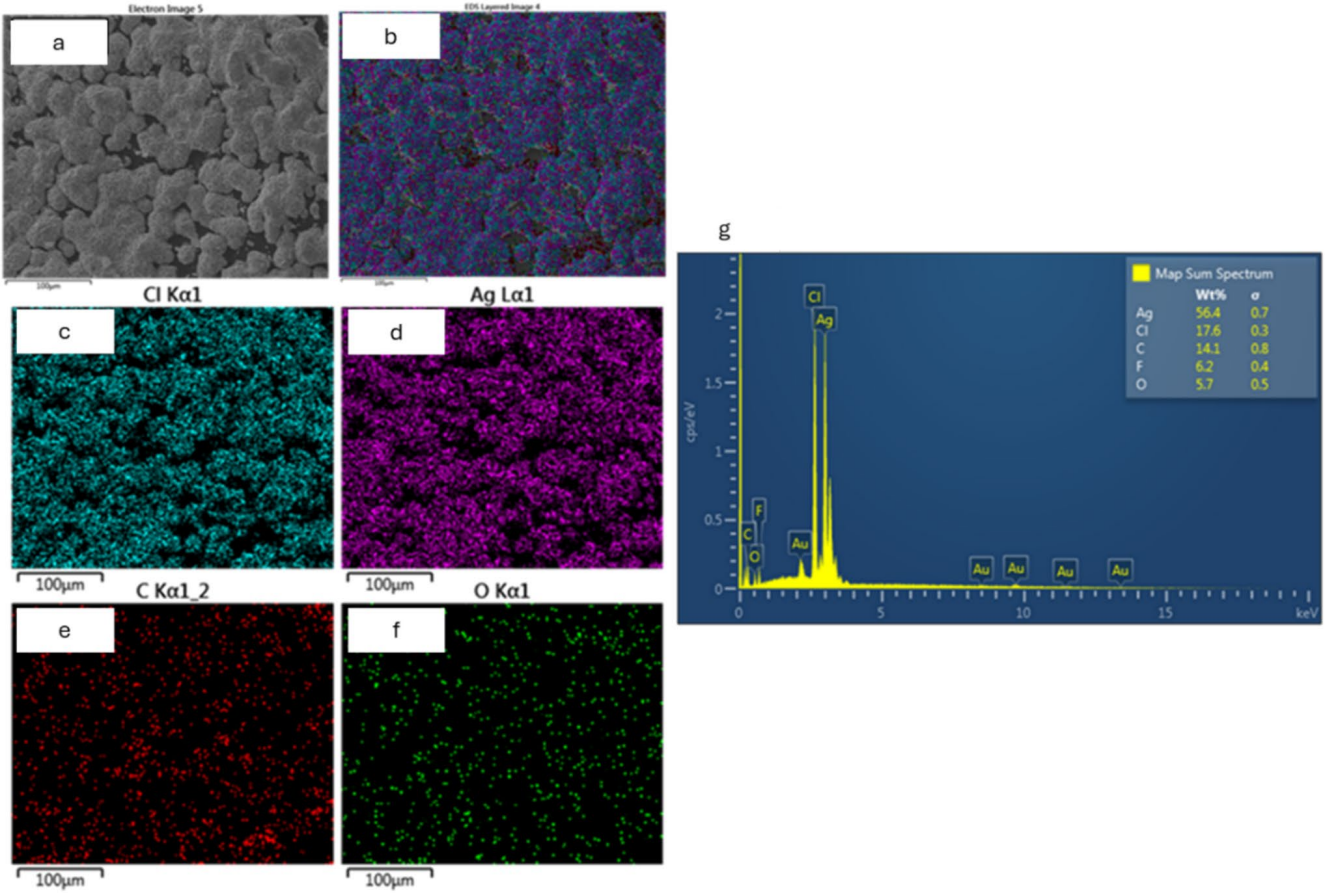
Membrane surface morphology (**a**), the corresponding EDS mappings (**b**–**f**), and spectra (**g**) of Ag-modified M-Ag4 membrane

**Fig. 5:**
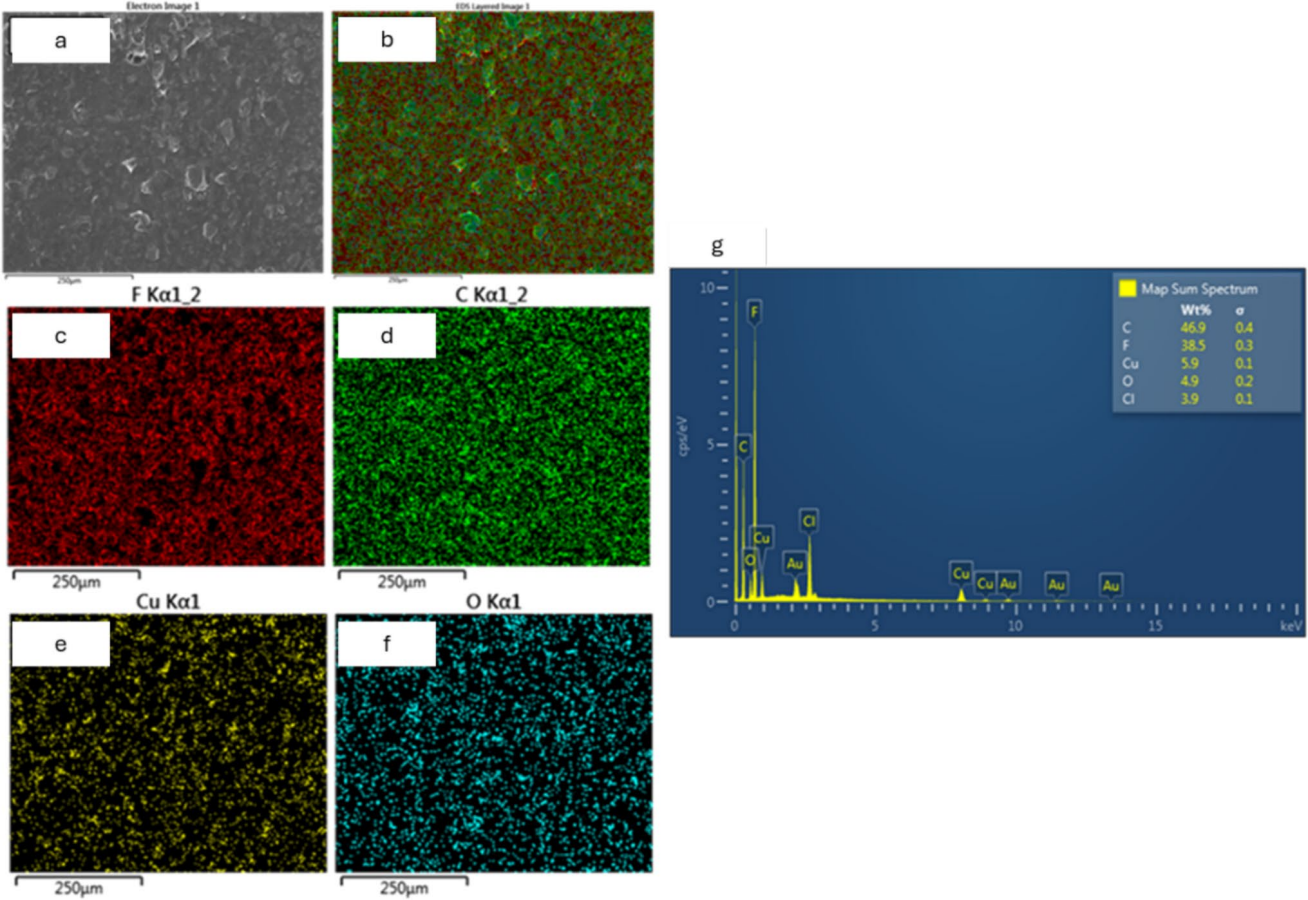
Membrane surface morphology (**a**), the corresponding EDS mapping (**b**–**f**), and spectra (**g**) of Cu-modified M-Cu4 membrane

EDS analysis confirmed that the immobilized nanoparticles embedded in the M-Ag4 and M-Cu4 surfaces consist of Ag and Cu, respectively. The EDS mapping of M-Ag4 and M-Cu4 surfaces showed that Ag and Cu NPs were homogenously distributed (Figs. 4 and 5). EDS spectra indicated that AgNPs are predominantly concentrated on the M-Ag4 surface with Ag content of 56.4% while C, Cl, and O had a lower content of 14.1 wt%, 17.6 wt%, and 5.7 wt%, respectively. A lower Cu content (5.9 wt%) is reported on the M-Cu4 membrane surface which is a further indication that CuNPs are largely embedded into the surface of the membrane. Also, the EDS spectra for both M-Ag4 and M-Cu4 showed the presence of Cl which indicates the quaternary ammonium functional group of the AEM have returned to the Cl phase.

#### Particle size determination

The average hydrodynamic diameters of Ag and Cu particles formed on modified membranes are presented in Table 2. The average hydrodynamic diameters of Ag for M-Ag1, M-Ag2, M-Ag3, and M-Ag4 were 167.7 ± 1.3 nm, 246.2 ± 2.9 nm, 499.6 ± 1.7 nm, and 652.2 ± 13.5 nm, respectively. These results indicated that the particle size increased with increasing concentration of the Ag precursor solution. As observed in the SEM images (Fig. 1b–e), the number of particles per unit area appears to increase with increasing concentration of the Ag precursor solution. Additionally, aggregation of particles was observed in SEM images for the Ag-modified membrane, resulting in particle sizes exceeding 100 nm.

**Table 2:** The average hydrodynamic diameter of Ag and Cu particles loaded on the modified membranes

Sample	Hydrodynamic particle diameter (nm)	
	Ag	Cu
M-Ag1	167.7 ± 1.3	
M-Ag2	246.2 ± 2.9	
M-Ag3	499.6 ± 1.7	
M-Ag4	652.2 ± 13.5	
M-Cu1		117.8 ± 2.0
M-Cu2		300.0 ± 1.2
M-Cu3		478.7 ± 30.0
M-Cu4		606.5 ± 9.6

Standard error corresponds to the standard deviation of three replicates of the membrane samples

For the Cu-modified membranes (Table 2), the average hydrodynamic diameters for Cu particles in the M-Cu1, M-Cu2, M-Cu3, and M-Cu4 membranes were 117.8 ± 2.0 nm, 300.0 ± 1.2 nm, 478.7 ± 30.0 nm, and 606 ± 9.6 nm, respectively. Like the Ag-modified membranes, the Cu particle size increased as the concentration of the Cu precursor solution increased. Moreover, evidence of aggregation of Cu particles was observed on the SEM image, which resulted in large particle sizes (Fig. 1k).

This study was intended to produce nanocomposite membranes using the IMS technique. However, it is clear from the hydrodynamic average diameters that the particles formed are larger than 100 nm. Taking into account that the actual particle size is smaller than the hydrodynamic diameter, the particle sizes obtained even from the lowest concentration of metal precursor solution are larger than 100 nm. The possible explanation for these results may be the excess number of Ag^+^ and Cu^2+^ due to the high concentration of the metal precursor solutions, which causes high surface energy and rapid particle growth (Alonso et al. 2013). At high surface energy, atoms have a strong attraction that causes particle agglomeration and hence large particle size (Ahmad et al., 2018). An alternative explanation is the high density of ion exchange sites per unit area, which promotes increased nucleation and growth of particles (Thanh et al. 2014). In this context, ion exchange sites serve as templates or seeds for structural formation, and a higher quantity of these sites results in a greater proliferation of structures (Tu et al. 2016).

#### Metal content analysis

The metal content of Ag and Cu-modified membranes was evaluated by immersing membrane pieces in concentrated HNO_3_ for 24 h to completely dissolve all the metal particles, and the results are shown in Table 3. The Ag content of M-Ag1, M-Ag2, M-Ag3, and M-Ag4 was 0.05 ± 0.003 mg·cm^−2^, 0.12 ± 0.004 mg·cm^−2^, 0.18 ± 0.01 mg·cm^−2^, and 0.17 ± 0.01 mg·cm^−2^, respectively. For the Cu-modified membranes, the metal content of M-Cu1, M-Cu2, M-Cu3, and M-Cu4 was 1.05 ± 0.004 mg·cm^−2^, 1.14 ± 0.005 mg·cm^−2^, 1.90 ± 0.02 mg·cm^−2^, and 2.13 ± 0.02 mg·cm^−2^, respectively. The metal content for both Ag and Cu-modified membranes was found to increase with increasing metal precursor solution from 0.01 M to 0.1 M. This result agrees with an earlier study by Alonso et al. (2013), wherein the metal content increased with increasing precursor solution concentration. The increase in metal content is attributed to the increased number of Ag^+^ and Cu^2+^ ions in the solution with increasing concentration (Ahmad et al., 2018). The obtained metal content results indicated that the amount of Cu loaded on the membrane matrix is more than that of Ag. The nature of the polymeric matrix has been reported to influence the amount of metal that is loaded (Alonso et al. 2013). This is attributed to the number of functional groups per unit area of the polymer matrix. However, in this case, the same polymer was used for all modified membranes. The differences in Ag and Cu metal loading onto the membranes can be attributed to the differences in ionic mobility of Ag^+^ and Cu^2+^. The mobility of ions in an electrolyte depends on the ionic radius (Saito et al. 2016). Thus, the large ions move slower than the small ions, and the Ag^+^ ionic radius (115 pm) is larger than that of Cu^2+^ (73 pm) (Banerjee & Bagchi 2019; Bastos-Arrieta et al. 2016a, b; Domènech et al. 2013). These results suggest that in the time frame the experiment was conducted more Cu^2+^ penetrated the surface of the membrane and was reduced by the borohydride resulting in a higher metal content.

**Table 3:** The Ag and Cu content in modified membranes

Sample	Metal content (mg·cm^−2^)	
	Ag	Cu
M-Ag1	0.05 ± 0.003	
M-Ag2	0.12 ± 0.004	
M-Ag3	0.18 ± 0.01	
M-Ag4	0.17 ± 0.01	
M-Cu1		1.05 ± 0.004
M-Cu2		1.14 ± 0.005
M-Cu3		1.90 ± 0.02
M-Cu4		2.13 ± 0.02

Error corresponds to standard deviation of three replicates of the membrane samples

### Metal-modified membrane stability

Nanocomposite stability experiments were performed to determine if the release of Cu and Ag was within the permissible levels for potable water. The leaching of Ag and Cu was monitored over 14 days, and the metal loss was determined on days 7 and 14 (Table 4). The rate of loss of Ag for the M-Ag1 membrane which has the least metal content was 5.02 ± 0.21% after 7 days and 6.21 ± 0.14% after 14 days, while the M-Ag4 membrane with the most Ag content had a rate of loss of 2.91 ± 0.05% after 7 days and 3.16 ± 0.07% after 14 days. The Cu nanocomposite membrane was more stable. The rate of loss for the M-Cu1 membrane with the least Cu content was 0.21 ± % after 7 days and 0.27% after 14 days. For M-Cu4, the rate of loss was 0.14% after 7 days and 0.25% after 14 days. It is noteworthy that the metal loss percentage does not denote the concentration of Ag and Cu in the water but the percentage of metal released from the original nanocomposite membranes. The highest loss for Ag and Cu was 6.62 ppb after 14 days and 5.32 ppb after 14 days from the M-Ag4 and M-Cu4 membranes, respectively. Moreover, the concentrations of Ag and Cu leachates (Table 4) also indicated that the leaching levels increased with increased metal content of the membranes.

**Table 4:** Metal leaching from Ag and Cu functionalized membranes under static conditions for 7 and 14 days

**Sample**		**Metal loss**		
	**7 days**		**14 days**	
	**ppb**	**(%)**	**ppb**	**(%)**
M-Ag1	2.51 ± 0.21	5.02 ± 0.21	3.11 ± 0.14	6.21 ± 0.14
M-Ag2	2.47 ± 0.12	2.39 ± 0.12	2.81 ± 0.08	2.79 ± 0.08
M-Ag3	3.78 ± 0.17	2.16 ± 0.17	4.67 ± 0.09	2.71 ± 0.09
M-Ag4	6.15 ± 0.05	2.91 ± 0.05	6.62 ± 0.07	3.16 ± 0.07
M-Cu1	2.15 ± 0.06	0.21 ± 0.06	2.75 ± 0.04	0.27 ± 0.04
M-Cu2	2.62 ± 0.02	0.23 ± 0.02	3.19 ± 0.02	0.28 ± 0.02
M-Cu3	2.66 ± 0.02	0.14 ± 0.02	2.85 ± 0.02	0.15 ± 0.02
M-Cu4	2.98 ± 0.03	0.14 ± 0.03	5.32 ± 0.04	0.25 ± 0.04

Three replicates of membrane samples were submerged in deionized water for 7 and 14 days

According to the WHO guidelines for drinking water, the permissible Ag and Cu thresholds are 0.1 mg·L^−1^ and 1.3 mg·L^−1^, respectively (US EPA, 2018; WHO, 2017). From the results obtained (Table 4), the membranes with the highest leaching concentrations could be used for disinfection since their leaching concentrations are below the threshold values. The metal loss percentages (Table 4) revealed that Cu-modified membranes were more stable than Ag-modified membranes. This observation is associated with morphology (Fig. 2) wherein Cu particles are embedded into the membrane surface, while the Ag particles are predominantly on the surface of the membrane, resulting in higher metal loss percentages. Also, the observed increase in leachate concentrations with increasing metal content can be attributed to the Ag and Cu particle size and particle agglomeration (Ashraf et al., 2018). The metal loss percentages of Ag and Cu over 14 days have revealed a relatively low-steady metal leaching rate, and the release of Ag and Cu is regarded as the leaching of metal ions (Ag^+^ and Cu^2+^) rather than the leaching of MNPs. These results indicate that the Ag and Cu NPs were stably loaded onto the membranes and can provide long-term antibacterial activity. Therefore, such nanocomposite membranes have the potential to be used as an emergency disinfectant in remote areas (Kwaadsteniet et al., 2011; Young & Santra, 2014).

### Bacteria inactivation by Ag and Cu-modified membranes

**Fig. 6:**
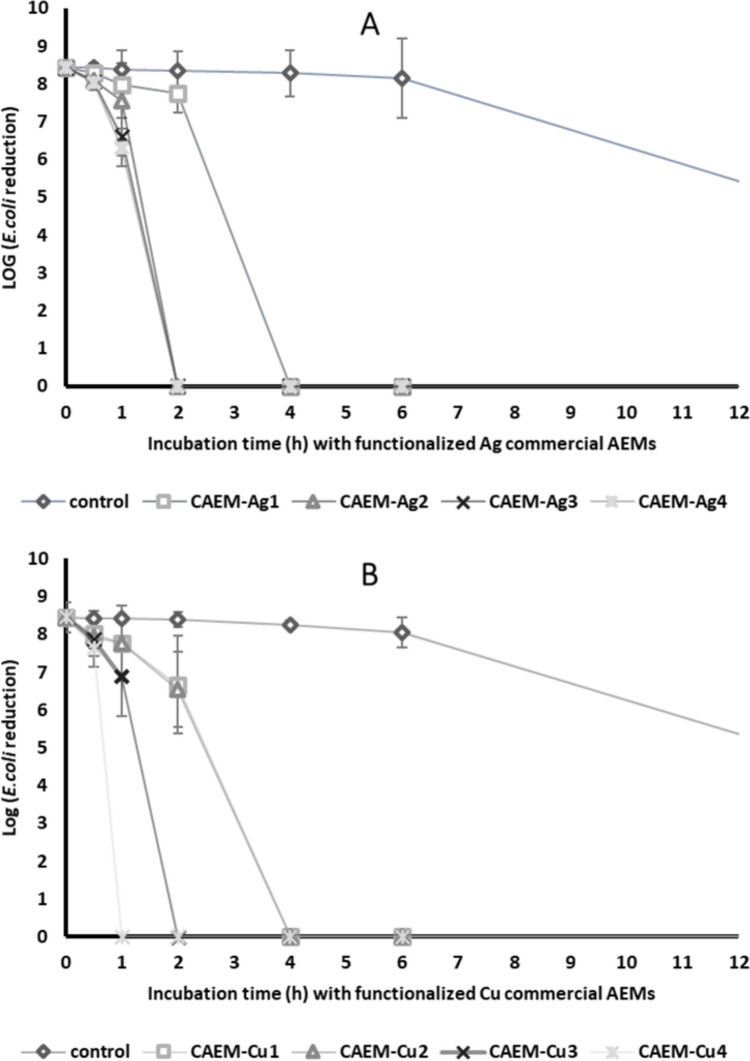
Log reduction of *E. coli* bacterial counts after a batch shake test conducted over time with pristine and metal-modified membranes: **a** pristine and Ag-modified membrane and **b** pristine and Cu-modified membrane featuring varying metal content loading. The initial concentration of bacteria was approximately 10^8^ CFU/mL

The bacteria inactivation by Ag and Cu functionalized membranes was evaluated using the indicator bacteria *E. coli*. Figure 6 shows the *E. coli* reduction during the inactivation tests with unmodified membranes and Ag and Cu functionalized membranes. The M-Ag4 with the most Ag metal content (0.17 ± 0.01 mg·cm^−2^) achieved an 8 log *E. coli* inactivation in 2 h, while the Ag membrane with the lowest amount of Ag (M-Ag1: 0.05 ± 0.003 mg·cm^−2^) took 4 h (Fig. 6a) to achieve similar levels of inactivation. For Cu, M-Cu4 (2.13 ± 0.02 mg·cm^−2^) was able to inactivate all the *E. coli* in 1 h and M-Cu1 (1.05 ± 0.004 mg·cm^−2^) in 4 h (Fig. 6b). A study done by Domenech et al. (2014) presented that Ag-modified SPESC and Nafion-117 inactivated *E. coli* in about 2 h with a metal content of 0.31 ± 0.01 and 5.9 ± 0.5 meq·cm^−2^, respectively. The unmodified membrane had no significant change in bacteria concentration in the first 6 h. After 6 h, a decay of bacteria in the control suspension was observed, although at a much-reduced rate. A possible reason for the decay may be due to the lack of nutrients in the media. These results suggest that the pristine membrane matrix has no antimicrobial activity. The inactivation of *E. coli* by Ag and Cu-modified membranes has been linked to the metal content of these membranes. The results from leaching tests demonstrated that metal ions were released from the membranes, with the quantity of these ions released dependent on the initial metal content within the membranes (see Table 4). As the leaching increased, so too did the rate of bacterial inactivation. Consequently, the inactivation observed with the modified membranes can be attributed to the release of metal ions and the contact mechanism by which bacteria are inactivated, a process that has been documented in previous studies (Alayande et al. 2020; Dankovich et al. 2016; Zikalala et al. 2023). The effectiveness of water treatment and disinfection processes is assessed using a concept known as “log removal values.” For pristine waters, a minimum of 4-log inactivation of bacteria is recommended, while an 8-log reduction is advised for heavily contaminated sources (Lau et al. 2024). The most efficient silver (Ag) and copper (Cu) membranes have shown an 8-log inactivation within 1–2 h.

To further evaluate the inactivation efficacy of the Ag and Cu functionalized membranes, the rate constant (*K*_d_) of inactivation was determined (Table 5). The calculated *K*_d_ for M-Ag1 at 0.5 h was 2.71 ± 0.20 (mg·cm^−2^)^−1^ which is approximately 7 times higher than that of M-Cu1 (0.39 ± 0.29 (mg·cm^−2^)^−1^) (Table 5). Similarly, with other membranes of higher metal loading, the inactivation rate of Ag was higher than Cu (Table 5). Although the Ag metal content was lower than that of Cu, it exhibited better specific antimicrobial activity. These results are associated with the morphology of Ag-modified membranes. The Ag particles are predominantly on the surface of the membrane, making them easily accessible. Furthermore, the metal leaching tests indicated relatively higher Ag release compared to Cu over time, which coincides with the greater inactivation rate. This outcome suggests that the leaching of metal ions is one of the mechanisms of *E. coli* inactivation.

**Table 5:** Kd values for *E. coli* inactivation of Ag and Cu-modified membranes

Sample	kd ((mg·cm^2^)^−1^)		
	0.5 h	1 h	2 h
M-Ag1	2.71 ± 0.20	9.04 ± 0.27	13.68 ± 0.54
M-Cu1	0.39 ± 0.29	0.65 ± 0.28	1.69 ± 0.08
Kd_Ag_:Kd_Cu_	7.74	13.91	8.09
M-Ag2	6.45 ± 0.43	7.41 ± 0.57	-
M-Cu2	0.58 ± 0.07	0.58 ± 0.06	1.67 ± 0.02
Kd_Ag_:Kd_Cu_	11.12	12.78	-
M-Ag3	4.82 ± 0.64	9.82 ± 0.27	-
M-Cu3	0.31 ± 0.24	0.83 ± 0.06	-
Kd_Ag_:Kd_Cu_	15.54	11.83	
M-Ag4	5.42 ± 0.06	12.46 ± 0.13	-
M-Cu4	0.38 ± 0.16	-	-
Kd_Ag_:Kd_Cu_	14.26	-	-

### Antibacterial activity

**Fig. 7:**
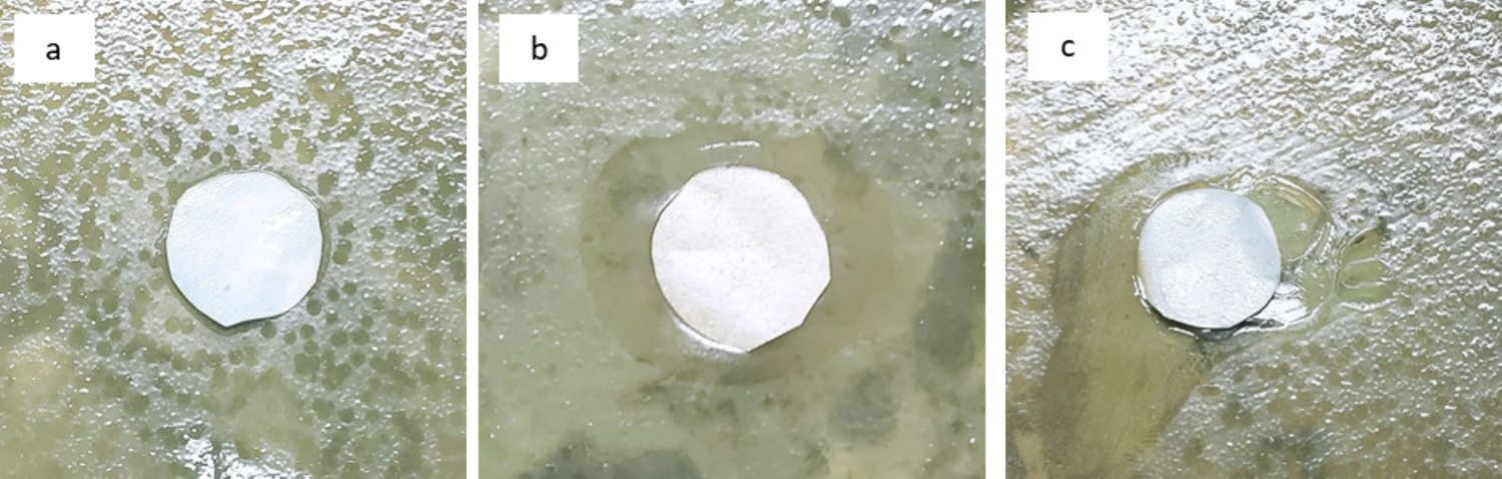
Zone of inhibition tests against *E. coli* using three different types of membranes: pristine membrane M-0 (**a**), Ag-modified membrane M-Ag-4 (**b**), and Cu-modified membrane M-Cu4 (**c**)

**Fig. 8:**
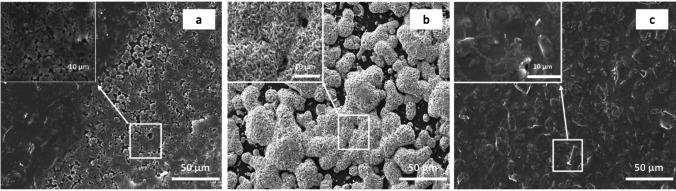
SEM micrographs of the pristine membrane M-0 (**a**), Ag-modified M-Ag4 (**b**), and Cu-modified membrane MCu4 after batch incubation with *E. coli* at 37 °C for 24 h

The antibacterial activity of the modified membranes was evaluated by the agar diffusion test as a surrogate of biofouling. This test showed that both the Ag and Cu-modified membranes inhibited the growth of *E. coli* (Fig. 7). The unmodified membrane did not show any inhibitory effect toward *E. coli*, as evidenced by bacteria growth around the membrane edge (Fig. 7a). Both the Ag and Cu-modified membranes had inhibition zones whereby the bacterial growth was inhibited (Fig. 7b, c). These indicated that antibacterial activity was mainly caused by Ag and Cu nanoparticles and not by the pristine membrane. The antibacterial property of the modified membranes was further confirmed by SEM images of the different membranes taken from the diffusion experiment. The surface of the pristine membrane was covered with a dense layer of bacteria (Fig. 8a). The Ag-modified membrane surface was relatively clean and free of bacteria growth (Fig. 8b). The Cu-modified membrane (Fig. 8c) surface appeared nearly free of bacterial growth with only a few bacteria observed. The difference in antimicrobial activity of Ag and Cu-modified membranes is based on the distribution as well as the positions of nanoparticles on the membrane matrix. The SEM images in Fig. 2 indicate that Ag nanoparticles are on the surface of the membrane whereas Cu nanoparticles are partially embedded in the surface. Therefore, the synthesized Ag-modified membrane had a greater surface area of metal for bacterial contact resulting in enhanced antimicrobial properties to that of the Cu-modified membrane.

## Discussion

A comparison of published studies on modifying ion exchange materials by the IMS technique for water disinfection with this study is discussed. Table 6 compiles significant findings from research focused on metal nanocomposite ion exchange polymers developed via the IMS method. As reported in earlier studies, the integration of the IMS technique with DEE facilitates the deposition of zero-valent metal nanoparticles near the membrane surface. Similar to these studies, the modified AMI 7001 membranes in this work displayed distinct distributions of Ag and Cu particles across their selective layer. Despite variations in polymer matrices across the literature, a common observation is the surface localization of metal particles, attributed to electrostatic interactions between the ion exchange sites and metal ions. In most IMS-based nanocomposites reported in the literature, those developed by Domenech et al. (2014), Alonso et al. (2013), Mudau et al. (2023), and Bekchanov et al. (2025) produce monodisperse nanoparticles in the 10–80 nm range. For those ion exchange materials, functional groups act as nanoreactors that limit particle growth and prevent aggregation, resulting in nanoscale particles with high surface reactivity and strong antibacterial activity. In contrast, the present study generated larger microscale Ag and Cu particles (118–652 nm), which are not expected for IMS. These results are attributed to the high density of active ion exchange sites per unit area, leading to active sites being positioned too closely together in AMI 7001. An elevated density of ion exchange sites increases nucleation opportunities and particle growth, ultimately leading to aggregation and formation of larger particles that exceed the nano-range, resulting in the production of a composite membrane rather than a nanocomposite as previously reported. The precursor solution’s metal ions also influence the composite membrane’s surface morphology. This study indicates that, based on ionic mobility and hydration radii, Cu particles were more integrated into the membrane surface, whereas Ag particles were predominantly located on the surface. This observation is attributed to the larger hydration radius of Cu ions, which facilitates faster permeability into the membrane matrix. Consequently, the positioning of Cu within the membrane allows for a higher content of copper to be incorporated during the modification process relative to Ag. Further, Cu-embedded particles exhibited a lower leaching percentage compared to Ag, which was more loosely attached to the membrane surface.

**Table 6:** Modified ion exchange polymers with antimicrobial activity

Metal	Polymer	Metal loading	Particle sizes/hydrodynamic diameter	Leaching rate	Microorganism (dose CFU/mL)	Inactivation time (h)	Log reduction (CFU)/inhibition zones (mm)	Reference
AgNPs	AMI 7001	0.05–017 mg·cm^−2^	652.2–167.7 nm	2.15–5.32 ppb	*E. coli* 15597 (10^8^)	1–4	8	This study
CuNPs	AMI 7001	1.05–2.13 mg·cm^−2^	606.5–117.8 nm	2.47–4.67 ppb	*E. coli* 15597 (10^8^)	1–4	8	This study
AgNPs	CEM AEM	0.020–0.034 mg·cm^−2^ 0.052–0.084 mg·cm^−2^	62.42–101.0 nm 89.4–121.1 nm	4.82–8.41 ppb 5.21–8.72 ppb	*E. coli 25922 (10*^*9*^*)* *E. coli 25922 (10*^*9*^*)*	0.5 0.5	5 5	(Mudau et al. 2023)
CuNPs	CEM AEM	0.031–0.062 mg·cm^−2^ 0.053–0.218 mg·cm^−2^	54.2–77.2 nm 79.8–125.7 nm	2.02–2.94 ppb 3.93–5.32 ppb	*E. coli 25922 (10*^*9*^*)* *E. coli 25922 (10*^*9*^*)*	0.5 0.5	5 5	(Mudau et al. 2023)
AgNPs	SPES-C٭ Nafion-117	0.3 meq·cm^−2^ 5.9 meq·cm^−2^	11.9 ± 0.2 nm 9.0 ± 0.2 nm	6.7%Ag/h 0.4%Ag/h	*E. coli* (10^6^)	2	6	(Domenech et al., 2014)
AgNPs	C100E˜ SST80^¥^ C104E˜ SST104^¥^	0.254–0.064^*^ 0.053^*^ 0.054–0.010^*^ 0.010^*^	13 nm	< 1 ppm Ag	*Pseudomonas putida (P. putida) KT2442 (10*^*5*^*)*	0.17–1	5	(Alonso et al. 2013)
Ag@Co NPs	C100E˜ SST80^¥^ C140E˜ SST104^¥^	0.069^*^ 0.061^†^ 0.053^*^ 0.073^†^ 0.011^*^ 0.017^†^ 0.010^*^ 0.017^†^	21 nm˜ 30 nm^¥^	< 1 mg·L^−1^ Ag l < 0.1 mg·L^−1^ Co	*P. putida KT2442 (10*^*5*^)	0.08–0.5	5	(Alonso et al. 2013)
AgNPs	Granulated resin A520E^9^ (Purolite)	0.11–0.57^*^	14.4 ± 0.3 nm	25% Ag	*E. coli*, CGSC 5073 K12 (10^5^) *P. putida*, KT2442 (10^4^)	Not reported	4	(Alonso et al. 2012a, b)
Ag@Fe_3_O_4_ NPs	Granulated resin A520E^9^ (Purolite)	1.7 mmol/meq Ag 1.1 mmol/meq Fe	Not reported	25% Ag@Fe_3_O_4_	*E. coli*, CGSC 5073 K12 (10^5^) *P. putida*, KT2442 (10^4^)	Not reported	4	(Alonso et al. 2012a, b)
Ag@Co§	FIBAN K-1 ^‡^ FIBANK-4^**¶**^	100–156 mg_Co_/g_NC_ 84–669 mg_Ag_/g_nc_ 323 mg_Co_/g_nc_ 469 mg_Ag_/g_NC_	8.8–14.4 nm	Not reported	Coliforms and Gram-positive bacteria (10^5^)	0.42–1	5	(Alonso et al. 2012a, b)
CuO NPs	Anion exchange (PPE-4)	Not reported	12.9–57.7 nm	Not reported	*E. coli*, MTCC443, Gram-positive (10^8^) *P. aeruginosa*, MTCC1688, Gram-negative (10^8^) *Staphylococcus aureus* (*S. aureus*) MTCC96, Gram-positive (10^8^)	Refrigerated at 4 °C (± 1 °C) for 2 h and incubated at 36 °C (± 1 °C) for 16–18 h	*E. coli* 26 mm *P. aeruginosa* 35 mm *S. aureus* 21 mm	(Bekchanov et al. 2025)

All studies were undertaken using lab-scale synthetic water except (§), which was tested using tap water, lab water, and river water

^*^ = sulfonated poly (ether sulfone); ˜ = granulated cation exchange matrix (carboxylated type); ¥ = granulated cation exchange matrix (sulfonated type); ‡ = sulfonated polypropylene fiber copolymerized with styrene and divinylbenzene); ¶ = carboxylated polypropylene fiber copolymerized with acrylic acid; * = mmol_Ag_/meq; † = mmol_Co_/meq

The findings of Bekchanov et al. (2025) provide important insight into how particle size influences antimicrobial performance in functionalized ion-exchange materials. In their study, incorporation of CuO nanoparticles in the 13–58 nm range resulted in large inhibition zones (21–36 nm) against *E. coli*, *Pseudomonas aeruginosa* (*P. aeruginosa*), and *Staphylococcus aureus *(*S. aureus*). This high antimicrobial activity was attributed to the large specific surface area of nanoscale CuO, which enhances generation of reactive oxygen species, metal–ion dissolution rates, and the likelihood of direct nanoparticle–cell interactions. The nanoscale dimensions also facilitate uniform distribution across the polymer matrix, improving contact efficiency with bacterial cells. By contrast, the Ag and Cu particles formed in the present work were significantly larger (118–652 nm) and exhibited varying degrees of aggregation. These larger particles inherently possess a lower reactive surface area per unit mass and lower dissolution rates, which would typically be expected to produce weaker antimicrobial effects. However, despite the larger particle size observed in this study, the modified membranes achieved complete inactivation of *E. coli* (8-log reduction) within 1–4 h, even at an initial concentration of 10^8^ CFU/mL. These results compare favorably example with earlier IMS studies, such as Domenech et al. (2014), where complete inactivation of 10^6^ CFU/mL was achieved in approximately 2 h. Difference in antimicrobial kinetics across the studies can also be attributed to experimental setup. Immersion or recirculation-based assays typically yield faster inactivation due to longer contact time, while filtration-based systems (e.g., Mudau et al 2023) exhibit lower log reductions because contact time is limited during permeation.

Overall, Table 6 shows that IMS is a versatile technique for introducing antimicrobial metals into ion exchange matrices. The findings of this study emphasize the influence of functional group density, precursor properties, and particle distribution on particle size, stability, and antimicrobial performance. Importantly, the results demonstrate that effective bacterial inactivation can occur even when IMS produces microscale particles, provided that sufficient contact time and controlled metal release are achieved.

## Conclusions

Modification of commercial anion exchange membranes using AgNO_3_ and CuSO_4_ through the IMS technique resulted in the formation of composite membranes with distinct metal–polymer interactions. SEM analysis showed that Ag particles were mainly concentrated on the membrane surface, transitioning from flat plate-like to spongy structures as the AgNO_3_ concentration increased from 0.01 M to 0.1 M. In contrast, Cu particles appeared primarily as flat structures embedded within the membrane surface. The hydrodynamic diameters of both Ag and Cu particles exceeded 100 nm, which indicated rapid particle growth driven by the high density of ion exchange sites in the pristine membrane. Increasing the precursor concentration from 0.01 M to 0.1 M led to higher metal loading, ranging from 0.05 to 0.17 mg cm^2^ for Ag and from 1.05 to 2.13 mg cm^2^ for Cu. Correspondingly, metal release into water increased, with Ag leaching between 0.31 and 0.66 mg L⁻^1^ and Cu between 0.26 and 0.53 mg L⁻^1^. Despite this increase, the leaching levels remained below WHO drinking water threshold. The modified membranes demonstrated strong antimicrobial activity against *E. coli* under static conditions, achieving up to an 8-log reduction within 1–4 h. The Ag composite membranes showed significantly faster specific inactivation rate, with a rate 14.26 times higher for Ag (M-Ag4, 5.42 ± 0.06 (mg·cm^−2^)^−1^) compared to Cu (M-Cu4, 0.38 ± 0.16 (mg·cm^−2^)^−1^). Agar diffusion tests further confirmed the antibacterial performance of both Ag and Cu composites, and SEM images revealed minimal bacterial attachment on the modified surface.

Overall, this study demonstrated that the IMS process enabled stable incorporation of Ag and Cu into the anion exchange membrane and produced metal particles with controlled ion release and effective antimicrobial activity. However, despite the stability of the metal deposits, the particles formed did not fall within the nanoscale range, indicating that IMS under the conditions tested was not sufficient to generate nanoparticles on this commercial membrane matrix. Nevertheless, the resulting composite membranes exhibited strong antimicrobial performance and therefore remain promising for decentralized or emergency water treatment applications where passive, material-based disinfection is beneficial.

## Data Availability

The data supporting this study are available from the corresponding author upon reasonable request.

## Declarations

### Clinical trial number

This is not applicable.
